# Evidence for the Higher Importance of Signal Size Over Body Size in Aposematic Signaling in Insects

**DOI:** 10.1673/031.011.0104

**Published:** 2011-01-12

**Authors:** Triinu Remmel, Toomas Tammarub

**Affiliations:** Department of Zoology, Institute of Ecology and Earth Sciences, University of Tartu, 46 Vanemuise Street, Tartu 51014, Estonia

**Keywords:** life history, predation, signal strength

## Abstract

To understand the evolution of warning coloration, it is important to distinguish between different aspects of conspicuous color patterns. As an example, both pattern element size and body size of prey have been shown to enhance the effectiveness of warning signals. However, it is unclear whether the effect of body size is merely a side effect of proportionally increasing pattern elements, or if there is an effect of body size *per se.* These possibilities were evaluated by offering different sized artificial caterpillars with either fixed or proportionally increasing aposematic color signal elements to wild great tits, *Parus major* L. (Passeriformes: Paridae). The birds' hesitation time to attack each “caterpillar” was used as a measure of the warning effect. The hesitation time showed a significant, positive size-dependence with the caterpillars whose pattern elements increased proportionally with their body size. In contrast, no size dependence was found in the larvae with fixed-size signal elements. Such a difference in mortality curves is consistent with the idea that pattern element size is a more important aspect than body size in enhancing a warning signal. Since no evidence of an effect of body size *per se* on signal efficiency was found, this study does not support the hypothesis that aposematic insects gain more from large size than cryptic ones.

## Introduction

To understand the evolution of warning coloration, characteristic of various unpalatable insects, it is essential to have an insight into predators' perception of the coloration of their prey. This is greatly enhanced by distinguishing different aspects of coloration and testing their relative importance in creating the warning effect. Such aspects may include the size (Forsman and Merilaita 1999; Lindström et al. 1999b), shape, color (Gamberale-Stille and Tullberg 1999; Gamberale-Stille and Guilford 2003; Ham et al. 2006; Aronsson and GamberaleStille 2008), and number of signal elements, as well as their symmetry (Forsman and Merilaita 1999; Forsman and Herrström 2004), contrast with adjacent colors (Prudic et al. 2007), conspicuousness in the environment (Gamberale-Stille 2001; Prudic et al. 2007; Stevens et al. 2008), and distinctiveness from other, palatable species (Puurtinen and Kaitala 2006; Merilaita and Ruxton 2007).

Furthermore, there are other prey characteristics beside coloration that affect signal strength in aposematic insects. Repellent odors (Rowe and Guilford 1999; Lindström et al. 2001) and movements (Hatle and Salazar 2001), as well as hairiness (Mappes et al. 2005), appear to function as warning signals independently of coloration. Other traits such as gregarious life style (Gamberale-Stille 2000; Hatle and Salazar 2001; Riipi et al. 2001) and, importantly, large body size (Gamberale and Tullberg 1996; Hunter 2000; Mänd et al. 2007) have been shown to enhance the warning effect in the presence of aposematic color signals.

This study specifically addresses the effect of prey body size, which is frequently suggested to be an important amplifier of warning signals it displays. In particular, Gamberale and Tullberg (1996) demonstrated that naive domestic chicks had stronger aversion towards larger instars of aposematic larvae of a lygaeid bug. Similarly, Mänd et al. (2007) found wildcaught great tits to have a greater aversion towards larger artificial caterpillars with proportionally larger warning color elements. However, these findings can be interpreted in different ways, as it is not unequivocally clear, which aspect of the warning signal has been measured in each particular case. First, the size of the conspicuous signal elements often increases with body size (as in many lepidopteran larvae, e.g. Sandre et al. 2007) and, therefore, might cause body size dependent differences in predation risk. There is, indeed, some evidence that signal element size affects the survival of aposematic insects. For example, Forsman and Merilaita (1999) showed that butterfly wing imitations with larger color spots were less attractive to domestic chicks. Large signal elements are also more effectively memorized by bird predators (Lindström et al. 1999b).

Alternatively, body size in aposematic insects could affect predators' aversion independently of the signal element effects (e.g. because predators might be more reluctant to consume large quantities of potentially toxic prey). This is indirectly supported by a finding that bird species with larger body size are more likely to eat aposematic baits (Exnerova et al. 2003). Additionally, the conspicuousness of prey increases with body size (Mänd et al. 2007) and conspicuousness as such can cause aversion in predators (Gittleman and Harvey 1980; Gamberale and Tullberg 1996). If body size *per se* is important, it may have substantial implications for the evolution of size, growth rate, and other size-related life history traits in aposematic species. Indeed, it has frequently been suggested that warningly colored insect species should benefit more than cryptic species from attaining large size (Forsman and Merilaita 1999, Hagman and Forsman 2003, Nilsson and Forsman 2003). The tendency of very small, early instar insect larvae to be cryptic rather than aposematic (Sandre et al. 2007) seems to support this idea.

However, the current knowledge about signal strength as a function of body size does not allow for differentiation between the relative impacts of signal element size and body size. In most insect species, color signal elements increase proportionally with body size (not necessarily for adaptive reasons). Because of this, it is hard to tell if the effect of body size is just a side-effect of signal element size or *vice versa.* Therefore, an experiment was conducted to explicitly compare these aspects, using artificial aposematic caterpillars as prey. Caterpillars of different body sizes and with either fixed size or proportionally increasing signal elements were offered to wild-caught birds, and the size-dependent “mortality” curves compared these two signal types.

## Methods and materials

To compare the relative importance of body size and the signal element size in determining the effectiveness of warning signal, artificial caterpillars of different body sizes and signal sizes were presented to wild birds. The artificial prey items were designed to imitate lepidopteran larvae with aposematic coloration. If and when the birds attacked each “caterpillar” was recorded.

### Predator species and artificial prey

Great tits, *Parus major* L. (Passeriformes: Paridae), were used as predators in the trials. This species is a common predator of herbivorous insect larvae in palearctic temperate forests. The bird experiments were carried out with the permission from the Estonian Ministry of the Environment. The birds were captured at Kabli Bird Station (SW Estonia) during the fall migration of 2004 (32 birds) and 2005 (35 birds). In captivity, they were kept in individual cages (80 × 80 × 80 cm) for up to 3 days and provided with sunflower seeds and fresh water *ad libitum.* The cages were illuminated during the natural daylight hours (i.e. between 11.5–12.5 hours per day). Prior to the experiment the birds were food deprived for about 2 h to increase their motivation to feed. Each bird was used only once, and released to the site of capture after the experiment.

Edible pastry (lard and flour, Church et al. 1997) cylinders were used as prey items. There were 7 size classes of such prey items: 2, 3, 4, 5, 6, 7, and 8 cm in length and about 0.25, 0.35, 0.5, 0.6, 0.75, 0.85, and 1 cm in width, respectively. The largest size classes corresponded to the largest caterpillars that occur in temperate areas; smaller larvae were not included because these are seldom found to display warning coloration in nature. The caterpillars were colored with black and yellow non-toxic finger paints; each larva had four dorsal yellow spots (or stripes in half of the cases in 2004, see below) on black background. Black was chosen for background and yellow for signal elements because larvae with classical black-yellow or black-orange warning coloration appear to be most repellent to birds when the bright colored area is maximized (Ojala 2006; Lindstedt et al. 2008; Lindstedt et al. 2009). The prey items were not flavored to be distasteful; however, the pastry caterpillars proved to be less favored food than mealworms or sunflower seeds and were never eaten more than a few bites, regardless of whether they were colored or not. Limited palatability of the test items ensured that the birds did not lose motivation to feed during the experiment, and allowed presentation of several prey items sequentially, without additional delay between each trial. All size classes were present in 2 signaling types: in the first type, the linear size of the yellow signal elements was proportional to body size, (i.e. the spot size increased with body length with diameters of about 0.25, 0.35, 0.5, 0.6, 0.75, 0.85, and 1 cm). In the second type, the signal element size was fixed (0.6 cm) for all size classes. The group with proportional-size signal elements henceforth will be called the *proportional-signal group,* and the group with fixed-size signal elements will be called the *fixed-signal group.* It would be desirable to vary body size and signal size independently, but since it was not possible to have large signal elements on small caterpillars, the two prey types described above were chosen.

A difference in the pattern of size-dependence of attack rate between the two types of prey items will allow us to evaluate if the body size effect can be ascribed to the increase in signal element size. In particular, if the acceptability of the prey items decreases in a similar manner in both signal types, it can be concluded that body size *per se* is the primary enhancer of warning signals. If, conversely, only the proportional-signal group displays a size dependent acceptability, it will be concluded that the warning effect is mostly dependent on the size of the signaling elements.

As a methodological detail, it must be noted that in 2004, the fixed-size signal elements were 0.6 cm wide stripes, not spots. The different signals in 2004 were used to test for the generality of body size dependent predation risk over different shapes of pattern elements. In 2005, the fixed-size signal elements were changed to be similar to those in the proportional-signal group, so that the effects of body size and signal size could be tested with pattern shape controlled for. However, the data from both years are pooled in the analysis, as all prey items in the fixed-signal group showed similar size-dependent predation curves, regardless of the shape of their pattern elements (see results).

### Experimental procedure

During the experiment, each bird was offered 7 pastry caterpillars (one of each size), one at a time in a random order. It was confirmed that the randomization process was successful as the presentation order was independent of larval size. Some of the birds received prey items with proportionally varying signal elements (N=29), and the rest received those with fixed-size signal elements (N=38). Each larva was offered to the bird together with a live mealworm (*Tenebrio molitor,* about 0.8 cm in length); eating the mealworm indicated that the bird was motivated to feed. Whether or not the bird attacked the larva within 10 minutes after eating the mealworm was recorded, and the hesitation time before attacking was measured (i.e. time elapsed from eating the mealworm until attacking the larva). After this, a new pair of prey (a mealworm and a pastry caterpillar) was offered immediately. If, however, the bird did not take either food item in 30 min, the experiment was suspended until the following day. The experimenter could view the inside of the cage through a small square of mesh without disturbing the bird; the prey were offered on a tray, which was pushed inside on a small drawer. The background was light beige, which rendered all prey items clearly visible but, most likely, did not interact with the coloration of the larvae in producing the warning effect.

### Data analysis

As the first step of the analysis, the probability of being attacked during the observation period (“fate”, as a binary variable) was modeled as dependent on *body size* (continuous), *signal type* (proportional or fixed-size signal elements), *year of experiment* (2004 or 2005), and *presentation order* (continuous) (logistic regression performed by SAS PROC GENMOD; SAS Institute Inc. 2007). Repeated measures analysis was applied to account for multiple (7) measurements on each individual bird. *Body size* was subtracted by its mean value (5 cm) to ensure that the statistics associated with main effects of categorical variables are interpretable for average sized caterpillars in the resulting heterogeneous slopes model (Littell et al. 2002). Squared value of *presentation order* was also included: a significant effect of the squared value of a numerical variable indicates a non-linear relationship between this variable and the response variable. In this analysis, a significant interaction between *signal type* and *body size* would indicate that size-dependence of repellence differed between the two signal types, which therefore constituted the effect of primary interest. Additionally, for illustrative purposes the signal types were analyzed separately, asking if *fate* depended on *body size.*

The logistic regression of *fate* as a binary variable does not, however, use the data in the most efficient way as it disregards the relevant information related to the hesitation time prior to attacking each larva, which is similarly a measure of repellence of the larva. However, logically, this measure was not available for prey items that were not attacked during the observation period (10 min). To allow for a combined analysis of all data, all such caterpillars were assigned hesitation time of 10 min. As a consequence, however, *hesitation time* could not be treated as a continuous variable, but it rather represents a multilevel ranked one: for example, the caterpillars attacked with a 5 minutes hesitation time were assigned rank 5, and those attacked at 10 minutes or never were assigned rank 10. Consequently, hesitation time was analyzed as a multinomial variable assuming *cumlogit* as the link function (SAS PROC GENMOD, SAS Institute Inc. 2007). The structure of the model was identical to that of the logistic regression, described above.

Additionally, an analogous analysis was performed with only the fixed-signal group to see if there was a difference between the two pattern element shapes (spots and stripes) used in consecutive years. *Rank* and *body size* were incorporated as continuous variables.

## Results

Of the 469 pastry caterpillars exposed, 226 (46%) were attacked by the birds, the rest were ignored during the 10 min observation period. Individual birds showed considerable variation in their behavior: many birds only attacked one or two of the presented prey items, while others attacked most caterpillars with minimal delay. The mean hesitation time was 2.0 (±2.5 SD) min, with the median at 1.0 min (excluding those larvae that were never attacked).

**Figure 1.**
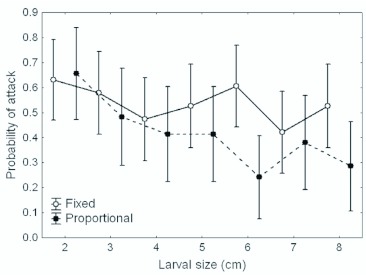
Means and 95% confidence limits for the probability of birds attacking different sized caterpillars. Open circles: caterpillars with fixed-size signal elements; closed circles: caterpillars with proportional-size signal elements. High quality figures are available online.

The probability of being attacked decreased as the larval size increased in the proportional-signal group (χ^2^ = 7.72, p = 0.0055) but was independent of body size in the fixed-signal group (χ^2^ = 1.00, p = 0.33) (Figure 1). However, a signal type × body size interaction, testing for a difference in size-dependence in the two groups, did not quite attain significance (χ^2^ = 2.85, p = 0.091).

When the power of the analysis was increased by analyzing the *hesitation time* before attack rather than the occurrence of attack alone (the multinomial analysis, see above), an interaction between signal type and body size was revealed which implies that the two groups showed different patterns of size-dependence (Table 1). While there was a significant main effect of year (the prey were somewhat more often attacked in 2004), there was no indication of a year × signal type (χ^2^ = 0.77, p = 0.38), or year × signal type × body size interaction (χ^2^ = 0.03, p = 0.98). This allows the among-year difference in the pattern element shape of the fixed-signal group to be disregarded, and the data of the two years to be combined. When analyzed separately, the striped and spotted caterpillars of the fixed-signal group did not significantly differ in the birds' hesitation times before attacking the prey items (χ^2^ = 3.73, P = 0.054), though the striped caterpillars in 2004 were attacked slightly more readily than the spotted ones in 2005. When analysing the two years separately the qualitative patterns remained similar, but the power of the analyses was reduced to the extent that interaction between body size and signal type could not be proved.

Both the order of presentation of a larva to a bird and the square of the presenting order had a strong positive effect on hesitation time (Table 1). In particular, the first presented caterpillars were attacked with 75% probability, and the last presented with 33% probability. However, this could not bias the main results as the presentation order was successfully randomized (i.e. it was independent of larval size) (one-way ANOVA: F(_6,462_) = 0.66, p = 0.68).

**Table 1.**
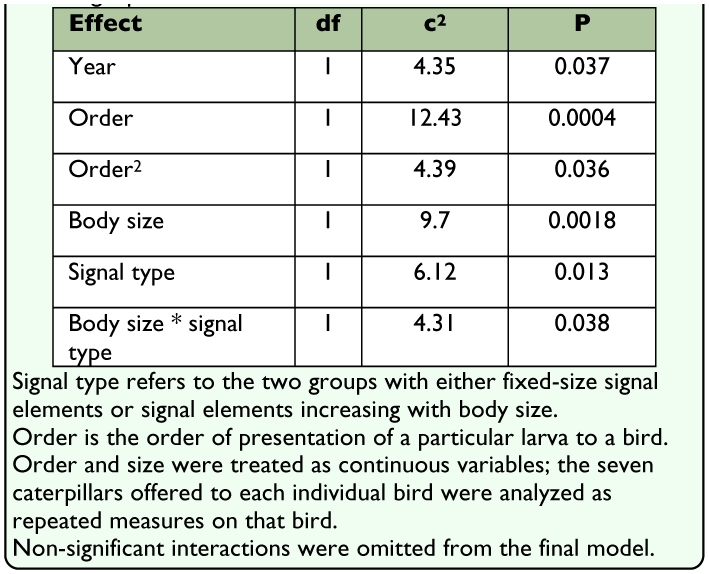
A multinomial model for birds' hesitation time before attacking a particular larva.

## Discussion

The increase in efficiency of the warning effect with body size was found only when signal size and body size were both increasing (Figure 1). This result confirms the idea that the size of aposematic signal elements affects the effectiveness of warning signals. However, no support was found for the effect of body size (independently of signal element size) on prey survival. The significant body size × signal type interaction implies that the different patterns of size-dependence in the two signal types could not be ascribed to chance.

Accordingly, results from this study suggest that body size as such is a less important amplifier of warning signals than the size of signal elements. This has important implications for the life-history evolution of aposematic insects: for example, it has been suggested that aposematic animals should benefit from growing larger than cryptic ones because their protection from predators is enhanced by large size (Forsman and Merilaita 1999; Hagman and Forsman 2003; Nilsson and Forsman 2003). The results of this study challenge the assumption of large body size *per se* enhancing the warning effect. However, a synergistic effect of body size combined with signal element size cannot be ruled out. It is plausible to suggest that even though body size had no demonstrable separate effect, it may still have contributed to the enhancement of the warning effect as the signal size increased. Moreover, the body size dependence of aposematic signal efficacy may be hard to overcome since several aspects of coloration may be correlated with body size, most obviously because very large (or perhaps numerous) signal elements cannot be displayed on very small animals.

A possible mechanism that may cause large signal elements to enhance the warning effect is the predators' biased generalization of signals towards larger element sizes. Such generalization biases can pose a selection pressure towards increasing signal strength in aposematic animals (Ruxton et al. 2009, Svádová et al. 2009). Alternatively, the emphasized avoidance of larger signal elements may be an innate trait in insectivorous birds.

The finding in this study that larger signal elements can substantially improve warning signals prompts one to ask why so many aposematic insects appear to display suboptimally small signal elements. The most plausible answer is that aposematism is often combined with crypsis (Endler 1978; Ruxton et al. 2004; Tullberg et al. 2005; Sandre et al. 2007). The conspicuous nature of a color pattern is a combined function of its signal element size and the viewing distance so that larger elements will appear conspicuous at relatively long distances, where smaller elements are still cryptic. Such compromise between aposematism and crypsis is favored in the cases in which predators differ in their acceptance of aposematic prey (Endler and Mappes 2004; Mappes et al. 2005; Speed and Ruxton 2007). The latter can result from variation in the predators' experiences, learning skills, ability to overcome prey defences, or hungriness (Exnerova et al. 2003; Exnerova et al. 2007; Sandre et al. 2007).

In prey preference experiments, it is often advisable to consider prey-to-background contrast as a trait that may add to the repellence of the prey (e.g. Gamberale-Stille 2001). Even though all prey items were clearly visible for the birds in this study and the background was selected to be neutral, it is still likely that larvae with larger yellow areas might have had lower contrast to the background. However, this apparently had little effect on the birds' choices. Had the birds preferred pastry larvae with lower contrast, one should expect that small *fixed-signal* larvae (with average size spots) were attacked more readily than small *proportional-signal* larvae (with small spots), and *vice versa* in the large size classes; however, the findings were quite the opposite. Consistently, other studies have demonstrated that birds tend to rely on prey color rather than contrast in assessing its profitability or in avoidance learning (Lindström et al. 1999a; Gamberale-Stille and Guilford 2003).

As a point of methodological significance, a strong effect of presentation order on the acceptability of prey items was found. The first presented caterpillars were accepted considerably more readily than the last presented ones. This may be primarily a result of the pastry caterpillars being a non-favored, though still palatable, food for great tits so that the birds learned to avoid them during the experiment. Most likely, the birds' hungriness also decreased during the experiment. Such a situation is reasonably natural, as warningly colored larvae are almost never preferred by predators and are only sampled when the predator is sufficiently hungry. The significant effect of the squared value of presentation order indicates that the process of learning and/or satiation slowed down towards the end of the experiment. Regardless of the above, since the presentation orders of different sized larvae were successfully randomized these effects cannot affect the qualitative findings concerning the efficiency of warning signals as dependent on body size or signal size.

In conclusion, the results of this study suggest that the body size dependence of signal strength can mainly be attributed to the effect of size-dependent change in signal element characteristics. However, other traits beside signal element size (e.g. the number of signal elements) which may also depend on body size, still remain to be tested. In any case, this finding illustrates the need to distinguish between different aspects of aposematic signals in order to understand the functioning of warning coloration.
